# Efficacy and Safety of Qingfei Paidu Decoction for Treating COVID-19: A Systematic Review and Meta-Analysis

**DOI:** 10.3389/fphar.2021.688857

**Published:** 2021-08-12

**Authors:** Qi Wang, Hongfei Zhu, Mengting Li, Yafei Liu, Honghao Lai, Qiuyu Yang, Xiao Cao, Long Ge

**Affiliations:** ^1^ Department of Social Medicine and Health Management, School of Public Health, Lanzhou University, Lanzhou, China; ^2^ Evidence Based Social Science Research Centre, School of Public Health, Lanzhou University, Lanzhou, China; ^3^ Evidence Based Nursing Centre, School of Nursing, Lanzhou University, Lanzhou, China; ^4^ WHO Collaborating Center for Guideline Implementation and Knowledge Translation, Lanzhou, China

**Keywords:** Qingfei Paidu decoction, traditional Chinese medicine, COVID-19, systematic review, meta-analysis

## Abstract

**Background:** Qingfei Paidu decoction (QFPD) has been widely used in treating COVID-19 in China. However, there is still a lack of comprehensive and systematic evidence to demonstrate the effectiveness and safety of QFPD. This study aims to evaluate the efficacy and safety of QFPD in patients with COVID-19.

**Methods:** We searched seven databases up to 5 March 2021. Two reviewers independently screened studies, extracted data of interest, and assessed risk of bias. The Cochrane risk of bias tool was used to assess the risk of bias of randomized controlled trials. The Newcastle–Ottawa scale was used to assess the risk of bias of cohort and non-randomized trials. The “Quality Assessment Tool for Before-After (Pre-Post) Studies With No Control Group” was adopted for controlled pre–post studies. We used the Grading of Recommendations, Assessment, Development, and Evaluations (GRADE) to assess the certainty of evidence. We carried out a random effect meta-analysis using RevMan 5.3. For outcomes that could not be meta-analyzed, we performed a descriptive analysis.

**Results:** We identified 16 studies with 11,237 patients, including one RCT, six non-randomized trials, two cohort studies, and seven pre–post studies. The certainty of evidence was low to very low because of the observational study design. QFPD combined with conventional treatment might decrease the time for nucleic acid conversion (MD = −4.78 days, 95% CI: −5.79 to −3.77), shorten the length of hospital stay (MD = −7.95 days, 95% CI: −14.66 to −1.24), shorten the duration of symptoms recovery of fever (MD = −1.51 days, 95% CI: −1.92 to −1.09), cough (MD = −1.64 days, 95% CI: −1.91 to −1.36) and chest CT (MD = −2.23 days, 95% CI: −2.46 to −2.00), improve the overall traditional Chinese medicine symptom scores (MD = 41.58 scores, 95% CI: 32.67 to 50.49), and change the laboratory indexes, such as WBC, AST, and CRP.

**Conclusion:** QFPD combined with conventional treatment might be effective for patients with COVID-19. No serious adverse reactions related to QFPD were observed. Further high-quality studies are still needed in the future.

## Introduction

Coronavirus disease 2019 (COVID-19) outbreak suddenly and quickly became a public health emergency of international concern (PHEIC) (World Health Organization, 2020), which has caused a pandemic and posed significant threats to international health. As of June 10, 2021, there have been 173.6 million confirmed cases of COVID-19, and 3.74 million deaths globally (World Health Organization, 2021b). As a newly discovered disease, the naming of this disease in traditional Chinese medicine (TCM) and Western medicine of COVID-19 is unified. It belongs to the category of “phytophthora blight” in TCM, which is a kind of disease caused by the epidemic pathogenic toxin with strong infectivity.

TCM, especially Chinese herbal medicine (CHM), contains thousands of years of health beliefs and practical experience in China (Fen et al., 2020) and has played and will continue to play an important role in the fight against the COVID-19 pandemic. Among the confirmed COVID-19 cases in China, 91.5% were treated with the combination of TCM and Western medicine, and the observation on clinical efficacy showed that the effective rate of TCM exceeded 90% (National Administration of Traditional Chinese Medicine, 2020).

Qingfei Paidu decoction (QFPD) is the only one that is recommended to treat all stages of COVID-19 by the Chinese national (General Office of National Health Committee, 2020) and local (Administration of traditional Chinese medicine of JiLin province, 2021) *Diagnosis and Treatment Protocol for Novel Coronavirus Pneumonia*. Almost all evidence-based guidelines and consensuses existed (Liang et al., 2020; Wang et al., 2020c), which strongly recommended QFPD treatment for patients with COVID-19. However, these recommendations were mainly based on indirect evidence and expert consensus and were not updated regularly. Up to now, several studies of QFPD have been published; however, there is still a lack of comprehensive and systematic review on the effectiveness and safety of QFPD.

To support our evidence-based guideline on integrating Chinese and Western medicine for COVID-19, we conducted this systematic review and meta-analysis to evaluate the effectiveness and safety of QFPD for patients with COVID-19 and assessed the certainty of evidence with the Grading of Recommendations, Assessment, Development, and Evaluations (GRADE) approach (Guyatt et al., 2008).

## Methods

This study was conducted and reported according to the Preferred Reporting Items for Systematic Reviews and Meta-Analyses (PRISMA) (Page et al., 2021). We prospectively registered this study protocol on the International Prospective Register of Systematic Reviews (PROSPERO: CRD42021233882).

### Eligibility Criteria

Inclusion criteria: 1) adults (age≥18 years) with COVID-19 of any severity who were confirmed by relevant diagnostic criteria (*Diagnosis and Treatment Protocol for Novel Coronavirus Pneumonia Trial Version 8* (General Office of National Health Committee, 2020)); 2) patients treated with QFPD or QFPD combined with Western medicine treatments; 3) patients in the control group who were given conventional support treatments (such as oxygen therapy, antiviral medications, or symptomatic therapies); 4) randomized controlled trials (RCTs), non-randomized controlled trials, cohort study, and controlled pre–post treatment studies.

Exclusion criteria: 1) the treatment group was a combined intervention of multiple Chinese medicines and the effect of QFPD cannot be obtained separately and 2) abstract, letter, theoretical discussion, commentaries, reviews, case reports, editorials, case–control studies, case series reports, and animal experiments.

### Search Strategy

We searched the WHO COVID-19 database, which included 26 databases published in different languages and gray literature evidence sources around the world (World Health Organization, 2021a), the Living Overview of the Evidence (L-OVE) COVID-19 Repository (Epistemonikos Foundation, 2021), PubMed, the China National Knowledge Infrastructure (CNKI), WanFang, the Chinese Biomedical Database (CBM), and the Chinese Medical Journal Network (Chinese Medical Journal Network, 2021). The search was performed initially on January 25, 2021, and updated on March 5, 2021. Search terms were “qingfei paidu decoction” or “qingfei paidu”. Any indexed terms equivalent to “QFPD” were also searched to extend the search coverage. There were no restrictions on publication language, year of publication, and publication status. The details of search strategies can be found in Supplementary Table 1.

### Study Selection and Data Extraction

Search records were imported into the reference management software Rayyan (Ouzzani et al., 2016). Two reviewers (HHL and YFL) independently screened the title and abstracts of each record and further reviewed the full texts of any potentially eligible studies for eligibility. Any disagreements were resolved by discussion or consultation with a third reviewer (LG).

A standard data extraction form was used to extract information from the included studies. Teams of two reviewers (ML and HZ, and QY and XC) extracted the data of interest including the first author, publication year, study design, sample size, patient type, age, sex, details of QFPD, dosage, treatment duration, control group, outcomes, adverse events, and other information. Another author (QW) double-checked the extracted data. Any conflict was resolved by discussion or adjudication by a third reviewer (LG) when necessary.

Combined with the core outcome sets of *COVID-19 Chinese medicine clinical research* (Jin et al., 2020), the outcomes we focused on included the time for nucleic acid conversion, the length of hospital stay, the TCM syndrome scores, the duration of symptom recovery, the effective rate, the rate of recovery of chest CT manifestations, the laboratory indexes (such as the biochemical indexes, the enzymology index, and the inflammatory factors), others (the disappearance rate of symptom recovery and mortality), and the incidence of adverse reactions.

### Risk of Bias Assessments

The risk of bias of RCTs was assessed by the Cochrane risk of bias (RoB) tool (Higgins et al., 2021). Each RCT was assessed at “low,” “high,” or “unclear” risk of bias according to seven domains: random sequence generation, allocation concealment, blinding of participants and personnel, blinding of outcome assessment, incomplete outcome data, selective outcome reporting, and other bias. The Newcastle–Ottawa scale (NOS) (Wells et al., 2012) was used to assess the risk of bias of cohort studies and non-randomized trials, which address eight questions in three broad categories: 1) patient selection; 2) comparability of study groups; and 3) assessment of the outcome. The maximum score of NOS was nine, and studies with scores of seven or more were graded as high quality while those with scores less than seven were considered low quality. The “Quality Assessment Tool for Before-After (Pre-Post) Studies With No Control Group” was adopted for controlled pre–post studies (NIH, 2014; Rosaria et al., 2021), which address twelve questions to be assessed at “yes,” “no,” or “other” (cannot determine, not applicable, or not reported) and each study was assessed at “good,” “fair,” or “poor” risk. Two reviewers (QW and HFZ) independently assessed the risk of bias for each study, and any disagreements were resolved by a third reviewer (LG).

### Certainty of Evidence Assessment

We assessed the certainty of evidence using the Grading of Recommendations, Assessment, Development, and Evaluations (GRADE) system (Guyatt et al., 2008), which classified evidence as to be high, moderate, low, or very low certainty. The starting point for the certainty for RCTs was high, and for observational studies was low. The certainty could be downgraded due to five reasons (risk of bias, imprecision, inconsistency, indirectness, and publication bias) and upgraded due to three reasons (large magnitude of an effect, dose-response gradient, and effect of plausible residual confounding).

### Data Synthesis and Statistical Analyses

We conducted random-effects model meta-analyses using the Review Manager software (RevMan, Version 5.3, Copenhagen: The Nordic Cochrane Centre, The Cochrane Collaboration, 2014). For dichotomous data, we calculated the risk ratio (RR) with corresponding 95% confidence interval (CI), and for continuous data, we calculated the mean difference (MD) with 95% CI. Missing data were imputed according to the Cochrane Handbook for Systematic Reviews of Interventions (van et al., 2003). Based on various study designs, we conducted meta-analysis separately if there were more than two studies. We also presented the results in the forest plot if there was only one study included. Statistical heterogeneity was assessed with *I* ^*2*^ statistic, and values of <25%, 25–50%, and >50% were considered as low, moderate, and high level of heterogeneity, respectively (Higgins et al., 2003). Egger’s test and funnel plots were used to detect the potential publication bias if the number of included trials was larger than ten. We performed subgroup analyses (e.g., severity of the disease) and sensitivity analyses to explore sources of heterogeneity if enough data were available.

## Results

**FIGURE 1:**
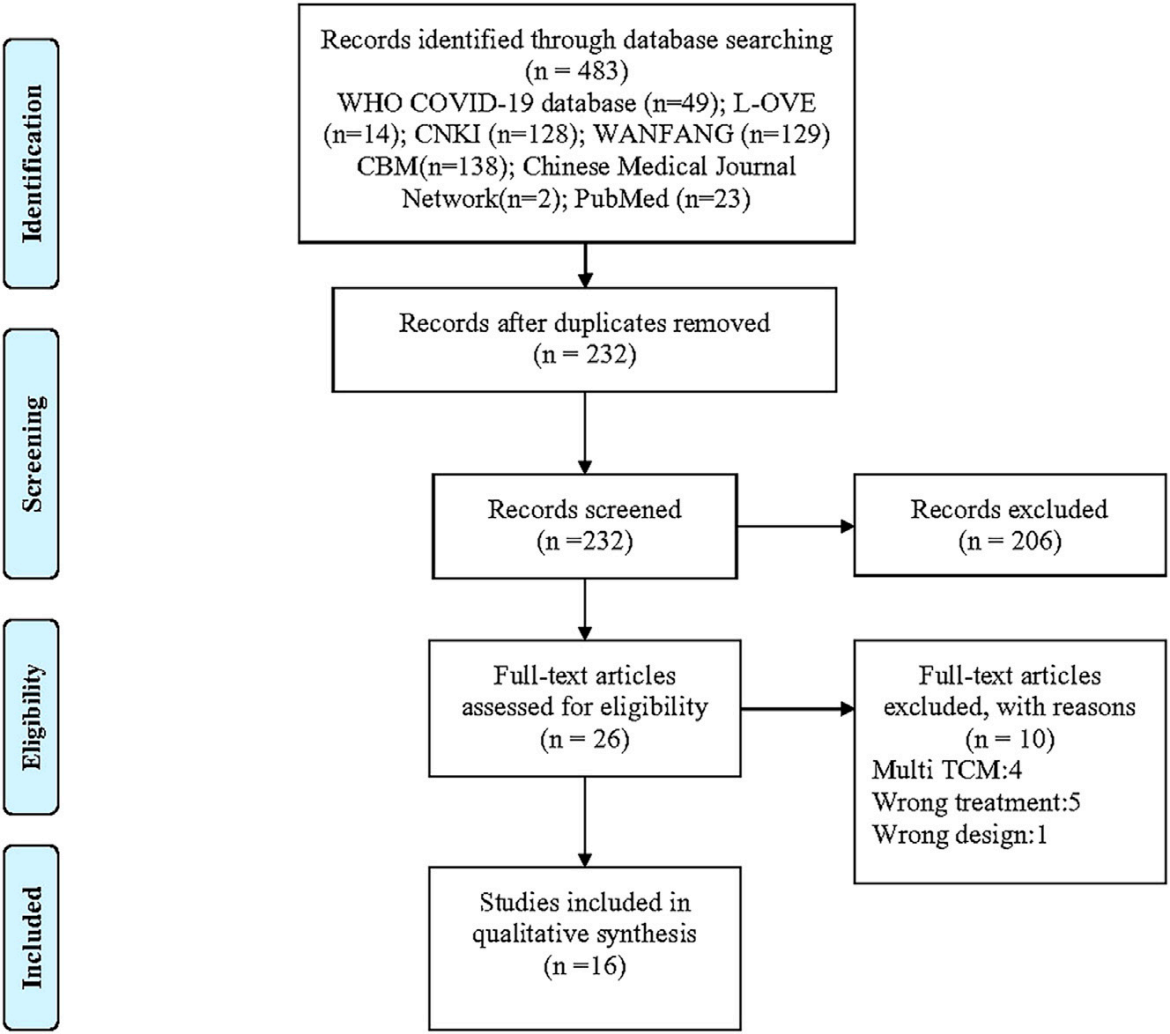
PRISMA flow diagram of study selection process.

The electronic searches yielded 483 unique studies. See Supplementary Table 2 for the reasons and lists of studies excluded in full-text screen. Finally, 16 studies proved eligible, which included one RCT, six non-randomized trials, two cohort studies, and seven pre–post studies (Figure 1). The summary table of included studies of QFPD is shown in Supplementary Table 3.

### Characteristics of the Included Studies

Table 1 shows the characteristics of the included studies. Sixteen studies (Dai et al., 2020; Hu et al., 2020; Li and Zhang, 2020; Li et al., 2020; Liu et al., 2020; Meng et al., 2020; Shi et al., 2020; Sun et al., 2020; Wang et al., 2020a; Wang et al., 2020b; Xin et al., 2020; Yu et al., 2020; Zhang et al., 2020a; Zhang et al., 2020b; Zeng et al., 2020) involved 11,237 patients (male: 47.26%) with COVID-19. All studies were conducted in China. The disease stages of COVID-19 were mild, moderate, severe, and critical. The mean age of patients in the included studies ranged from 40.5 to 62.3 years’ old. The durations of QFPD treatment ranged from 6 to 15 days.

**TABLE 1:** Characteristics of included studies.

Study	Study design	COVID-19 stage	Duration	Age (M ± SD)	Sample size	Male (*n*, %)	Treatment	Control
Li and Zhang (2020)	RCT	Severe: 100%	8d	NR	12	7 (58.33)	QFPD + C	Symptomatic treatments, antibiotic, antiviral treatment
Zhang et al. (2020a)	Cohort study	NR	NR	55.90 (15.60)	8,939	4,168 (46.63)	Patients receiving QFPD	Patients not receiving QFPD
Shi et al. (2020)	Cohort study	Non-severe: 91%; severe: 9%	NR	46.00^$^	782	405 (51.79)	QFPD + antiviral drugs, antibiotics, corticosteroids, α-IFN inhalation, and symptomatic treatments	NA
Xin et al. (2020)	Non-randomized trial	Mild and moderate	6d	51.57 (52.49)	63	29 (46.03)	QFPD + C	Oxygen therapy, antipyretic, rehydration, nutritional support, antiviral treatment, antibiotic, corticosteroids
Zeng et al. (2020)	Non-randomized trial	Moderate: 100%	NR	46.41 (5.92)	229	124 (54.15)	QFPD + C	Antiviral treatment, oxygen therapy, psychological intervention, health education
Li et al. (2020)	Non-randomized trial	NR	NR	52.02 (0.31)	60	28 (46.67)	QFPD + C	Nutritional support, respiratory support, antiviral treatment
Sun et al. (2020)	Non-randomized trial	Mild: 1.3%; moderate: 97.6%	NR	49.20 (13.68)	295	117 (39.66)	QFPD	QFPD+ antiviral treatment, antibiotic, Chinese patent medicine+ symptomatic treatments
Yu et al. (2020)	Non-randomized trial	Moderate: 34.8%; severe and critical: 65.2%	1 dose/d, bid, 10-15d	62.30 (2.23)	89	39 (43.82)	QFPD + C	Western medicine
Zhang and Pan (2021)	Non-randomized trial	Severe: 75.00%; critical: 25.00%	bid; 7d	62.84 (14.69)	24	12 (50.00)	QFPD + C	Antiviral treatment, anti-infection treatment, vitamin C, symptomatic treatments, α-IFN inhalation
Dai et al. (2020)	Pre–post study	Mild: 5.56%; moderate: 38.89%; severe: 55.55%	1 dose/d, bid; 6d	55.67 (16.21)	36	23 (63.89)	Western medicine + QFPD	None
Hu et al. (2020)	Pre–post study	Mild: 23.7%; moderate: 63.1%; severe and critical: 13.2%	bid; 15d	40.56 (15.01)	76	40 (52.63)	QFPD + C	None
Liu et al. (2020)	Pre–post study	NR	1 dose/d; bid; 7-14 d	40.50 (10.80)	13	4 (30.77)	Antiviral treatment, anti-infection treatment, oxygen therapy + QFPD	None
Meng et al. (2020)	Pre–post study	Moderate: 75.93%; severe: 24.07%	NR	60.08 (18.65)	108	41 (37.96)	Antiviral treatment, antibiotic, symptomatic treatments + QFPD	None
Wang et al. (2020a)	Pre–post study	Mild: 37.3%; moderate: 62.7%	1 dose/d, bid; 6 d	40.53 (16.60)	75	46 (61.33)	Western medicine + QFPD	None
Wang et al. (2020b)	Pre–post study	Mild: 55.10%; moderate: 33.67%; severe and critical: 11.22%	1 dose/d, bid; 9 d	42.70 (16.86)	98	52 (53.06)	Western medicine + QFPD	None
Zhang et al. (2020b)	Pre–post study	Mild: 9.47%; moderate: 17.75%; severe: 72.78%	1 dose/d; bid; 9 d	NR	338	176 (52.07)	Antiviral treatment, symptomatic treatments + QFPD	None

C, control; d, day (s); M, Mean; N, number; NA, not applicable; NR, not report; RCT, randomized controlled trial; SD, standard deviation; $, median.

We included 13 studies for quantitative analysis, which compared the combination of QFPD and conventional treatment to conventional treatment. One study (Sun et al., 2020) compared the combination of QFPD and conventional treatment to QFPD alone; we described this study qualitatively. Two retrospective cohort studies (Shi et al., 2020; Zhang et al., 2020a) could not be pooled with other studies; therefore, we described them qualitatively.

### Risk of Bias of Included Studies

The included RCT had serious risk of bias due to unclear risk of bias in random sequence generation, allocation concealment, blinding of participants and personnel, and blinding of outcome assessment (Supplementary Figure S1). Six non-randomized studies and two cohort studies were assessed using NOS; only three studies (Shi et al., 2020; Sun et al., 2020; Zhang et al., 2020a) showed “good” quality (Supplementary Table 4). The assessment results of seven pre–post studies showed that four studies (Dai et al., 2020; Hu et al., 2020; Wang et al., 2020b; Zhang et al., 2020b) were of “fair” quality and three studies (Liu et al., 2020; Meng et al., 2020; Wang et al., 2020a) were of “poor” quality (Supplementary Table 5).

### Outcomes

#### Time for Nucleic Acid Conversion

**FIGURE 2:**
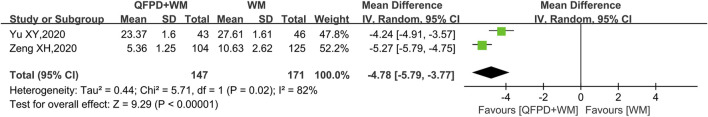
Meta-analysis result of the time for nucleic acid conversion.

Three non-randomized studies reported on the time for nucleic acid conversion. The pooled results of two studies (Yu et al., 2020; Zeng et al., 2020) showed that compared with conventional treatment, a significant reduction of the time for nucleic acid conversion was found for the combination with QFPD (MD = −4.78 days, 95% CI: −5.79 to −3.77; very low certainty) (see Figure 2; forest plot). Another non-randomized study (Sun et al., 2020) showed that the median time for nucleic acid conversion (10 days) in the combined group was significantly longer than the QFPD alone group (5 days) (*p* < 0.05) (low certainty of evidence).

#### Length of Hospital Stay

**FIGURE 3:**
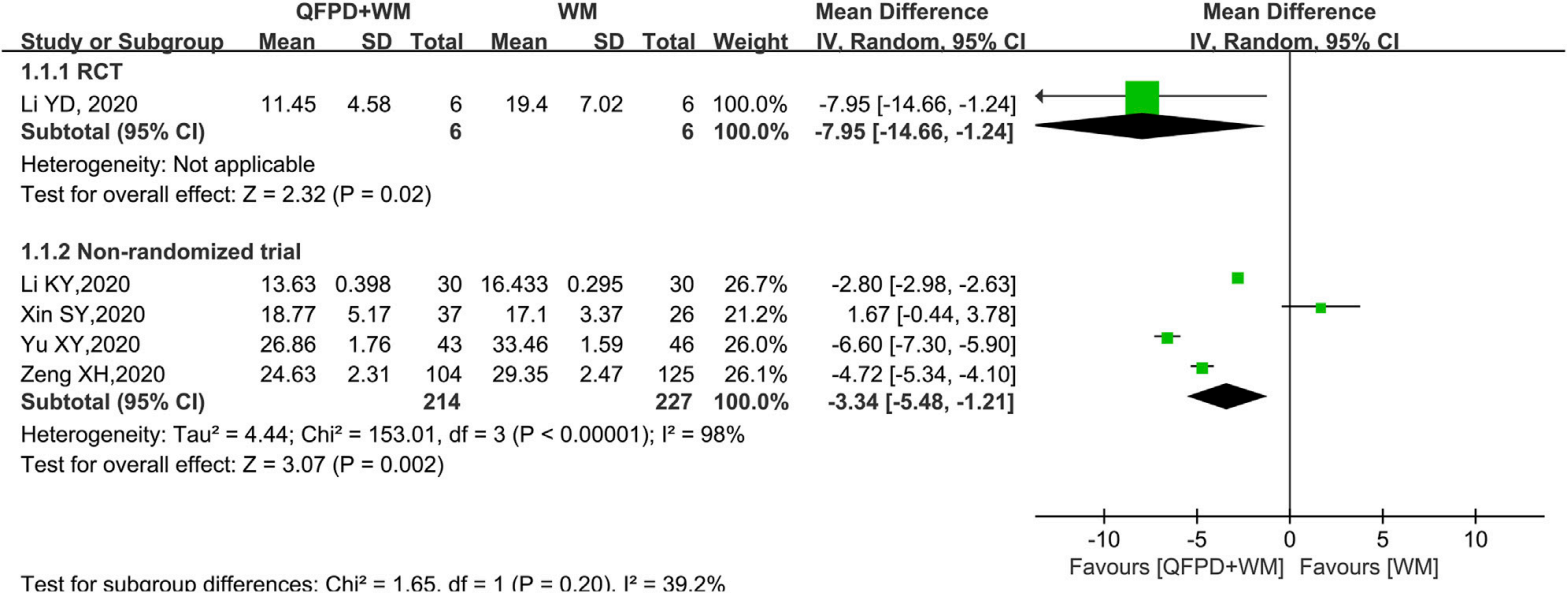
Meta-analysis result of the length of hospital stay.

Both of one RCT (Li and Zhang, 2020) (MD = −7.95 days, 95% CI: −14.66 to −1.24; low certainty) and the pooled results of four non-randomized trials (Li et al., 2020; Xin et al., 2020; Yu et al., 2020; Zeng et al., 2020) (MD = −3.34 days, 95%CI: −5.48 to −1.21; very low certainty) showed that the combination with QFPD could significantly shorten the length of hospital stay compared with conventional treatment (see Figure 3; forest plot).

A cohort (Shi et al., 2020) showed that treatment within a week was significantly associated with a reduction in the median duration of hospital stay of 1–4 days compared with later treatment (*p* < 0.0001). A non-randomized study (Sun et al., 2020) also found that the median length of hospital stay (16 days) in a combined group was significantly longer than the QFPD alone group (9 days) (*p* < 0.05).

#### TCM Symptom Scores

Two pre–post studies (Hu et al., 2020; Wang et al., 2020b) reported on the TCM symptom scores (see Supplementary Figure 2; forest plot). The pooled results showed that compared with pretreatment, QFPD could significantly improve the overall TCM symptom scores (MD = 41.58 scores, 95%CI: 32.67–50.49), cough (MD = 4.34 scores, 95%CI: 0.97–7.71), fatigue (MD = 4.06 scores, 95%CI: 0.21–7.91), anorexia (MD = 3.46 scores, 95%CI: 0.04–6.89), complexion (MD = 2.70 scores, 95%CI: 0.76–4.64), insomnia (MD = 1.43 scores, 95%CI: 0.05–2.81), hyperhidrosis (MD = 1.74 scores, 95%CI: 0.14–3.34), and urine (MD = 1.48 scores, 95%CI: 0.41–2.55). The certainty of evidence was very low.

Wang et al. (2020b) also found that QFPD could significantly improve the TCM symptom scores of expectoration (MD = 5.41 scores, 95%CI: 3.13–7.69), rhinobyon (MD = 7.08 scores, 95%CI: 4.52–9.64), runny nose (MD = 6.68 scores, 95%CI: 4.30–9.06), dry mouth (MD = 5.23 scores, 95%CI: 2.69–7.77), sore throat (MD = 4.08 scores, 95%CI: 1.36–6.80), palpitation (MD = 4.59 scores, 95%CI: 1.88–7.30), aversion to cold (MD = 5.93 scores, 95%CI: 3.44–8.42), cyanosis (MD = 10.13 scores, 95%CI: 7.05–13.21), short of breath (MD = 4.30 scores, 95%CI: 2.06–6.54), tongue picture (MD = 3.64 scores, 95%CI: 1.80–5.48), and pulse condition (MD = 2.16 scores, 95%CI: 1.07–3.25).

#### Duration of Symptoms Recovery

Compared with conventional treatment, the combination with QFPD could significantly shorten the duration of symptom recovery such as fever (MD = −1.51 days, 95%CI: −1.92 to −1.09; very low certainty), cough (MD = −1.64 days, 95%CI: −1.91 to −1.36; very low certainty), and chest CT (MD = −2.23 days, 95%CI: −2.46 to −2.00; very low certainty) (Li et al., 2020) (see Supplementary Figure 3; forest plot).

#### Effective Rate

The result of meta-analysis showed that compared with after 3 days of treatment, patients treated by QFPD after 9 days had a higher effective rate of TCM symptoms (Wang et al., 2020b), such as fever (RR = 1.10, 95%CI: 1.02–1.19), cough (RR = 1.10, 95%CI: 1.01–1.21), expectoration (RR = 1.37, 95%CI: 1.13–1.66), runny nose (RR = 1.16, 95%CI: 1.03–1.30), fatigue (RR = 1.14, 95%CI: 1.02–1.28), short of breath (RR = 1.16, 95%CI: 1.02–1.32), dry mouth (RR = 1.14, 95%CI: −1.01–1.28), insomnia (RR = 1.19, 95%CI: 1.08–1.30), anorexia (RR = 1.10, 95%CI: 1.00–1.23), complexion (RR = 1.11, 95%CI: 1.01–1.21), aversion to cold (RR = 1.13, 95%CI: 1.02–1.24), hyperhidrosis (RR = 1.34, 95%CI: 1.14–1.57), urine (RR = 1.11, 95%CI: 1.01–1.21), tongue picture (RR = 1.16, 95%CI: 1.06–1.26), and pulse condition (RR = 1.20, 95%CI: 1.08–1.33) (see Supplementary Figure 4; forest plot). The certainty of evidence was very low.

#### Laboratory Indexes

Compared with conventional treatment, the results of RCT (Li and Zhang, 2020) showed that the combination with QFPD had a significant improvement of WBC (MD = −4.47×10^9^/L, 95%CI: −7.12 to −1.82; low certainty), PCO_2_ (MD = 8.86 mmHg, 95%CI: 3.23 to 14.49; low certainty), and PO_2_ (MD = −20.80 mmHg, 95%CI: −34.59 to −7.01; low certainty) (see Supplementary Figure 5; forest plot).

The pooled results (Xin et al., 2020; Yu et al., 2020) of non-randomized trials found that QFPD could significantly improve the biochemical indexes (see Supplementary Figure 6; forest plot), such as AST (MD = 1.12U/L, 95%CI: 0.25–1.99), SCr (MD = 2.67 μmol/L, 95%CI: 2.05–3.29), and eGFR (MD = −2.28%, 95%CI: −2.82 to −1.74), and also could improve the level of cellular immunity of CD3, CD4, and CD8 (Yu et al., 2020) (see Supplementary Figure 7; forest plot).

The result of pre–post studies revealed that compared with the pretreatment, the combination with QFPD could improve the enzymology index of ALT, AST, and HBDH, and also improve the expression of inflammatory factors, such as CRP (MD = 20.00 μg/L, 95%CI: 15.90–24.11) and ESR (MD = 10.27 mm/h, 95%CI: 5.39–15.15) (see Supplementary Figures 8, 9; forest plot).

In addition, compared with after 3 days of treatment, patients treated with QFPD after 9 days had a higher recovery rate of laboratory indexes (Wang et al., 2020b) (see Supplementary Figure 10; forest plot), such as WBC (RR = 1.14, 95%CI: 1.05–1.25), NEUT (RR = 1.08, 95%CI: 1.01–1.15), CRP (RR = 1.16, 95%CI: 1.04–1.30), LYMPH (RR = 1.16, 95%CI: 1.04–1.30), ESR (RR = 1.30, 95%CI: 1.15–1.47), DD (RR = 1.08, 95%CI: 1.01–1.15), ALT (RR = 1.12, 95%CI: 1.01–1.24), and AST (RR = 1.12, 95%CI: 1.03–1.22).

#### Other Outcomes

We found that the combination with QFPD could improve the rate of recovery of chest CT manifestations (Zeng et al., 2020; Zhang and Pan, 2021) (RR = 1.26, 95%CI: 1.10–1.43) (see Supplementary Figure 11; forest plot). Besides, QFPD could improve the disappearance rate of symptom (Liu et al., 2020) (see Supplementary Figure 12; forest plot), such as fever, cough, expectoration, sore throat, fatigue, insomnia, spontaneous sweating, and irritability and anxiety. In addition, a non-randomized study (Sun et al., 2020) showed that after treatment, the disappearance time of sputum symptom in the combined group (median = 6 days) was significantly longer than that in the QFPD group (median = 2 days, *p* = 0.046), and the improvement of chest CT in the QFPD group was better than that in the combined group (*p* < 0.05).

For the recovery of COVID-19, a cohort (Shi et al., 2020) revealed that compared with treatment initiated after 3 weeks, early treatment with QFPD after less than 1 week, 1–2 weeks, or 2–3 weeks had a higher likelihood of recovery, with adjusted hazard ratio (HR) of 3.81 (95%CI: 2.65–5.48), 2.63 (95%CI: 1.86–3.73), and 1.92 (95%CI: 1.34–2.75), respectively.

For the in-hospital mortality, the result of a cohort (Zhang et al., 2020a) indicated that the crude mortality was 1.2% (95% CI: 0.8–1.7%) among patients receiving QFPD and 4.8% (95% CI: 4.3–5.3%) among those not receiving QFPD. After adjustment for patient characteristics and concomitant treatments, the use of QFPD was associated with a relative reduction of 50% in in-hospital mortality (HR = 0.50; 95% CI: 0.37–0.66). The certainty of evidence was low.

#### Incidence of Adverse Reactions

Hu et al. (2020) found that the rate of overall adverse reactions of QFPD was 5.3% (76 patients), including diarrhea, nausea and vomiting, skin itch, and symptoms were mild. Wang et al. (2020a) reported that the rate of adverse reactions of QFPD was 0.07% (98 patients), including nausea and vomiting, dizziness, and rash. Zhang et al. (2020b) found that 16.57% patients had hyperhidrotic, 5.92% epigastric pain, and 3.25% diarrhea. Other studies found that during the treatment, the occurrence of nausea (Li et al., 2020) and skin itch (Li and Zhang, 2020) were unrelated to QFPD.

#### Publication Bias

We did not assess the publication bias because of the limited number of studies.

## Discussion

COVID-19 belongs to the category of “phytophthora blight” in TCM. Damp-heat lung plague caused by damp-heat and epidemic toxin are the most widely accepted explanation of COVID-19 (Zhou, 2020). It is necessary to comprehensively understand according to the actual situation of the patient, especially considering the age, physique, disease status, treatment process, medication effect, and underlying diseases, and make dialectical differentiation of syndrome, disease, and treatment based on the doctor’s clinical experience. It can be divided into three main types—damp-toxin epidemic, cold-damp epidemic, and damp-heat epidemic (General Office of National Health Committee, 2020; Ren et al., 2020).

QFPD is not made up of TCM materials but multiple concordant prescriptions, including Maxing Shigan decoction, Shegan Mahuang decoction, Xiaochaihu decoction, and Wuling powder, which contribute to the symptomatic efficacy of QFPD. It is a Chinese formula, which comprises of 21 herbs: má huáng (*Ephedra sinica* Stapf) 9g, zhì gān cǎo (*Glycyrrhiza uralensis* Fisch.) 6g, xìng rén (*Prunus armeniaca* L. var.ansu Maxim.) 9g, shí gāo (Gypsum Fibrosum) 15–30 g (decocted first), guì zhī (*Cinnamomum cassia* Presl) 9g, zé xiè (*Alisma orientale* (Sam.) Juzep.) 9g, zhū líng (*Polyporus umbellatus* (Pers.) Fries) 9g, bái zhú (*Atractylodes macrocephala* Koidz.) 9g, fú líng (*Poria cocos* (Schw.)Wolf) 15g, chái hú (*Bupleurum chinense* DC.) 16g, huáng qín (*Scutellaria baicalensis* Georgi) 6g, jiāng bàn xià (*Pinellia ternate* (Thunb.) Breit.) 9g, shēng jiāng (*Zingiber officinale* Rosc.) 9g, zǐ wǎn (*Aster tataricus* L. f.) 9g, kuǎn dōng huā (*Tussilago farfara* L.) 9g, shè gān (*Belamcanda chinensis* (L.) DC.) 9g, xì xīn (*Asarumsieboldii* Miq.) 6g, shān yào (*Dioscorea opposita* Thunb.) 12g, zhĭ shí (*Citrus aurantium* L.) 6g, chén pí (*Citrus reticulata* Blanco) 6g, and huò xiāng (*Pogostemon cablin* (Blanco) Benth.) 9g. It possesses the treatment principle of clearing away heat and toxic material, reconciling the cardinal mechanism, eliminating phlegm and dispelling masses, dispersing blood stasis and dredge collateral, and meanwhile attaching great importance to the protection of lung function.

A systems pharmacological study (Zhao et al., 2020) investigated the mechanisms of QFPD against that of COVID-19 from the levels of molecule, pathway, and network, which revealed that the 88 high-confidence targets of the 12 active compounds in QFPD were overlapped with genes affected by COVID-19 infection. Through the comprehensive network and pathway analysis, the study demonstrated that QFPD possessed five functional modules including immune regulation, anti-infection, anti-inflammation, and multi-organ protection, which is attributed to the multi-component, multi-target, and multi-pathway characteristics of QFPD.

Based on the theory of TCM for syndrome differentiation and treatment, the mild case includes cold-damp constraint in the lung pattern and damp-heat accumulation in the lung pattern; the moderate case can be classified into damp-toxin constraint or cold-damp obstructing the lung pattern; the severe case is divided into epidemic toxin blocking the lung pattern and blazing of both qi and ying patterns; and the critical case is the internal blockage and external desertion pattern (General Office of National Health Committee, 2020). Liu et al. (2020) focused on patients with damp-toxin constraint in the lung pattern, which were manifested as fever, common in low fever, dry cough, less phlegm, bad throat, fatigue, poor appetite, dark tongue or slightly red edge, thick and greasy fur on the tongue, and few pulses. The basic prescription of QFPD needs to be modified with the symptoms. Meanwhile, most of the interventions in the studies we included were QFPD combined with Western medicine. In clinical application, Western medicine treatment should be used in accordance with the latest diagnosis and treatment guidelines. Symptomatic treatment is the major treatment for mild cases, antibiotics and systemic corticosteroids are not recommended. For patients with moderate COVID-19, pneumonia treatment should be treated as principal method, and antibiotics should be used if there is clinical suspicion of a bacterial infection. Severe cases are mainly treated for severe pneumonia treatment, and systemic corticosteroids are recommended for severe and critical patients (World Health Organization, 2021c). QFPD is suitable for patients with all stages of COVID-19, as is the combination of QFPD and Western medicine.

In addition, the previous study has confirmed that TCHM combined with Western medicine may improve clinical symptoms and shorten the length of hospital stay for SARS patients (Liu et al., 2006). This review can also reach such a conclusion that QFPD may be used as an effective adjuvant treatment for COVID-19.

QFPD has become a widely accepted prescription for treating COVID-19 because of its successful and effective clinical observations in China. The successful use of QFPD has confirmed the advantages of TCM in treating emergencies (Ren et al., 2020). On March 2, 2021, The National Medical Products Administration (NMPA) in China granted market approval of QFPD granules through special procedures (The National Medical Products Administration (NMPA), 2021).

We systematically assessed the efficacy and safety of QFPD treatment for patients with COVID-19, including 16 clinical trials with 11,237 patients. We found that the combination with QFPD could significantly decrease the time for nucleic acid conversion; shorten the length of hospital stay; improve the TCM symptom scores; shorten the duration of symptoms recovery such as fever, cough, and chest CT; and change the laboratory indexes such as WBC, AST, and CRP. No serious adverse reactions were found. These evidences revealed the important role of QFPD in treating COVID-19.

So far, there has been no systematic review on QFPD treatment of patients with COVID-19. Our review was performed with strict acceptance criteria and used the GRADE approach to assess the certainty of evidence. We included all types of study design to document the benefit and harm of QFPD. The results showed that for patients with COVID-19, QFPD combined with conventional treatment was probably better than conventional treatment alone. Therefore, our study will provide a comprehensive evidence support for QFPD in the treatment of patients with COVID-19. In addition, we used *the core outcome sets of COVID-19 Chinese medicine clinical research* and also focused on the important outcomes of patients, which could be more valuable and informative for decision-makers to use QFPD for the treatment of COVID-19. Meanwhile, our review was designed to support the development of an evidence-based guideline of COVID-19.

Our review has some limitations. First, all the studies included were from China, and the sample size for most of them was less than 100. Therefore, the present findings might not fully reflect the global situation and should be interpreted with caution. Second, due to the lack of methodological rigor in the included studies, the certainty of evidence was low or very low. Third, due to the limited number of studies included, we were not able to conduct subgroup analysis and disclose publication bias.

## Conclusion

With low to very low certainty of evidence, the combination of QFPD and conventional treatment may be effective in decreasing the time for nucleic acid conversion, improving the TCM symptom scores, shortening the length of hospital stay, reducing the duration of symptoms recovery, and improving the laboratory indexes. No serious adverse reactions related to QFPD were identified. More high-quality multicenter researches are still needed to further corroborate the effectiveness and adverse events of QFPD in the treatment of COVID-19.

## Data Availability

The original contributions presented in the study are included in the article/Supplementary Material; further inquiries can be directed to the corresponding author.

## Glossary

ALT: alanine aminotransferase
AST: aspartate aminotransferase
CBM: Chinese biomedical database
CHM: Chinese herbal medicine
CNKI: China National Knowledge Infrastructure
COVID-19: coronavirus disease 2019
CRP: c-reactive protein
CT: computed tomography
DD: D-dimer
eGFR: estimated glomerular filtration rate
ESR: erythrocyte sedimentation Rate
GRADE: the grading of recommendations, assessment, development, and evaluations
HBDH: hydroxybutyrate-dehydrogenase
HR: hazard ratio
L-OVE: the living overview of the evidence
LYMPH: lymphocyte
MD: mean difference
NEUT: neutrophil count
NMPA: The National Medical Products Administration
NOS: Newcastle-Ottawa scale
PCO_2_: partial pressure of carbon dioxide
PHEIC: Public Health Emergency of International Concern
PO_2_: partial pressure of oxygen
PRISMA: preferred reporting items for systematic reviews and meta-analyses
QFPD: Qingfei Paidu decoction
RCTs: randomized controlled trials
RoB: risk of bias
RR: risk ratio
SCr: serum creatinine
TCM: traditional Chinese medicine
95%CI: 95% confidence interval
WBC: white blood cell
