# Supplementary figures and images for: Efficacy and Safety of Qingfei Paidu Decoction for Treating COVID-19: A Systematic Review and Meta-Analysis
Source: Front Pharmacol. 2021 Aug 12;12:688857. doi: 10.3389/fphar.2021.688857 (PMC8387832; doi:10.3389/fphar.2021.688857)

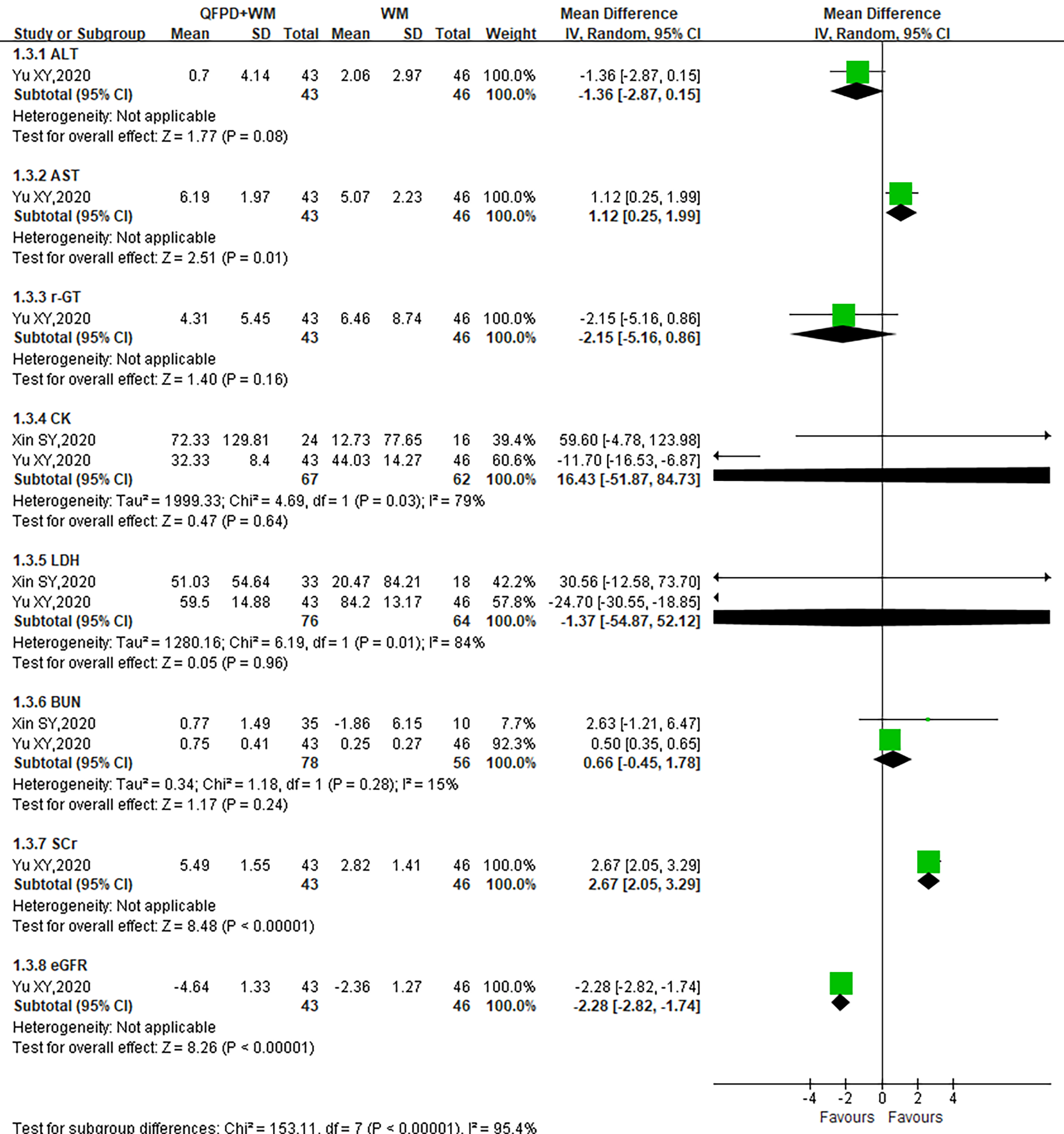

Supplement: Supplementary file 1 [file Image6.TIF]

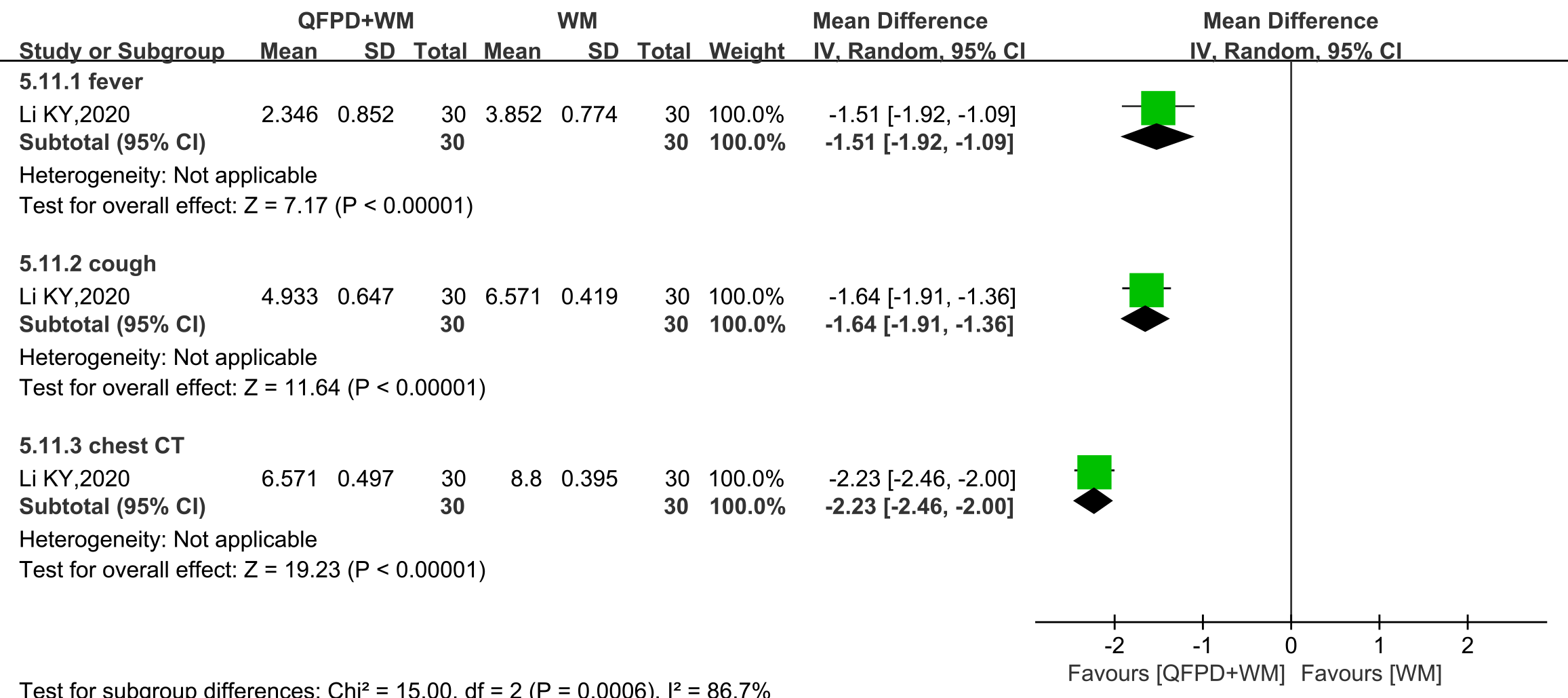

Supplement: Supplementary file 3 [file Image3.TIF]

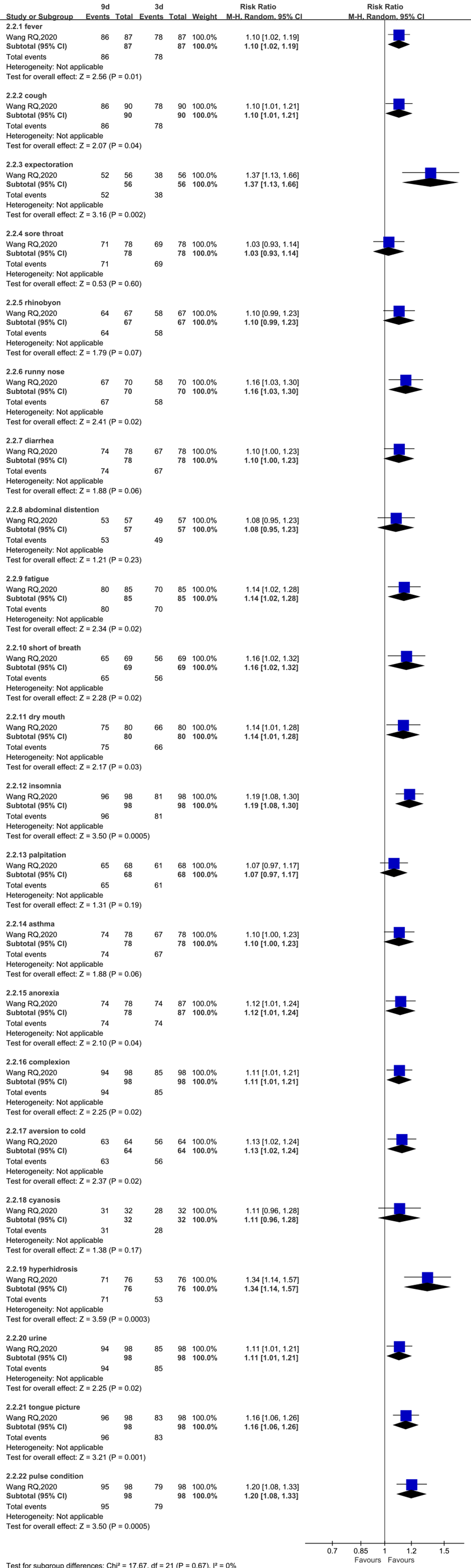

Supplement: Supplementary file 4 [file Image4.TIF]

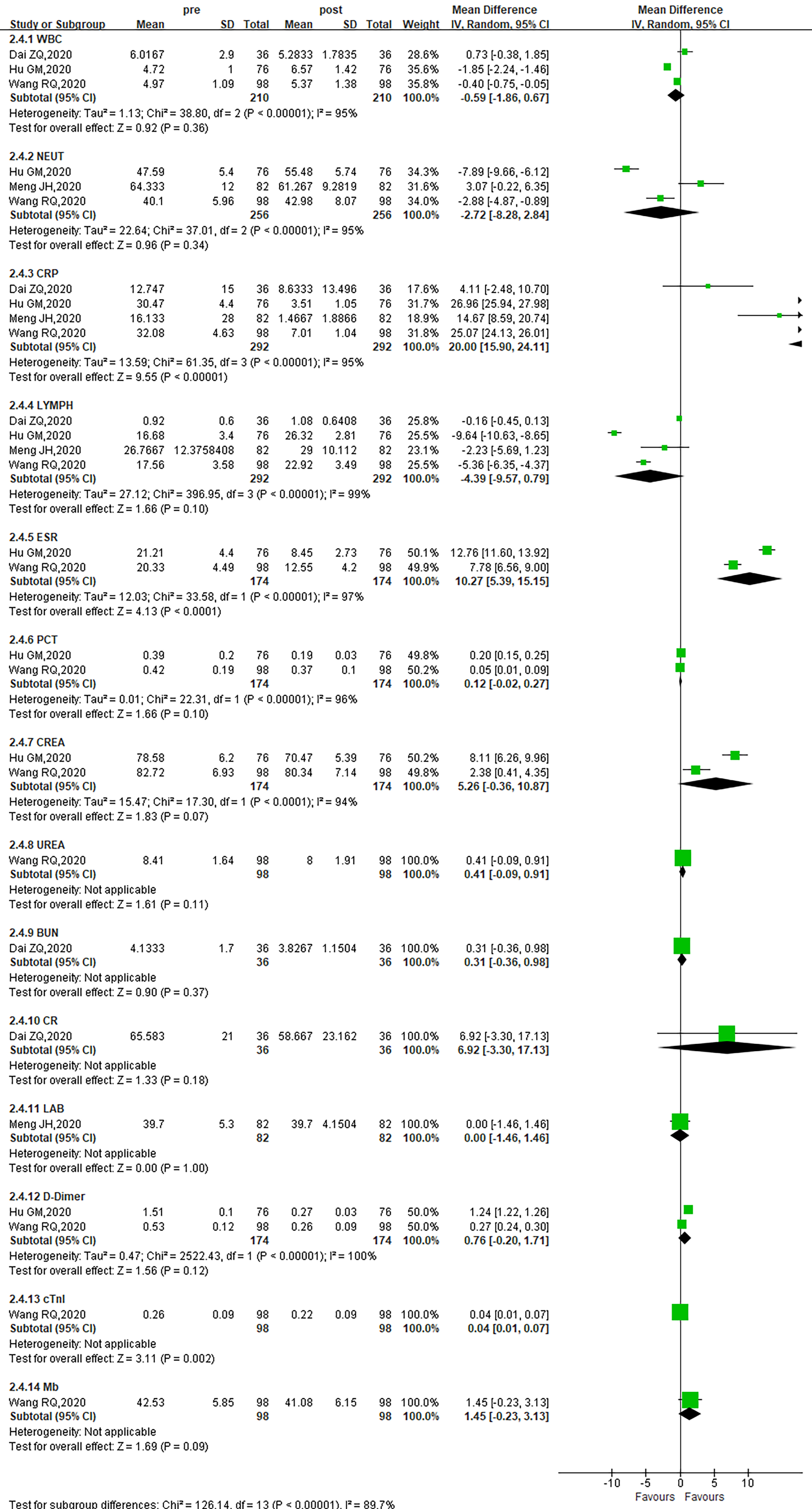

Supplement: Supplementary file 5 [file Image9.TIF]

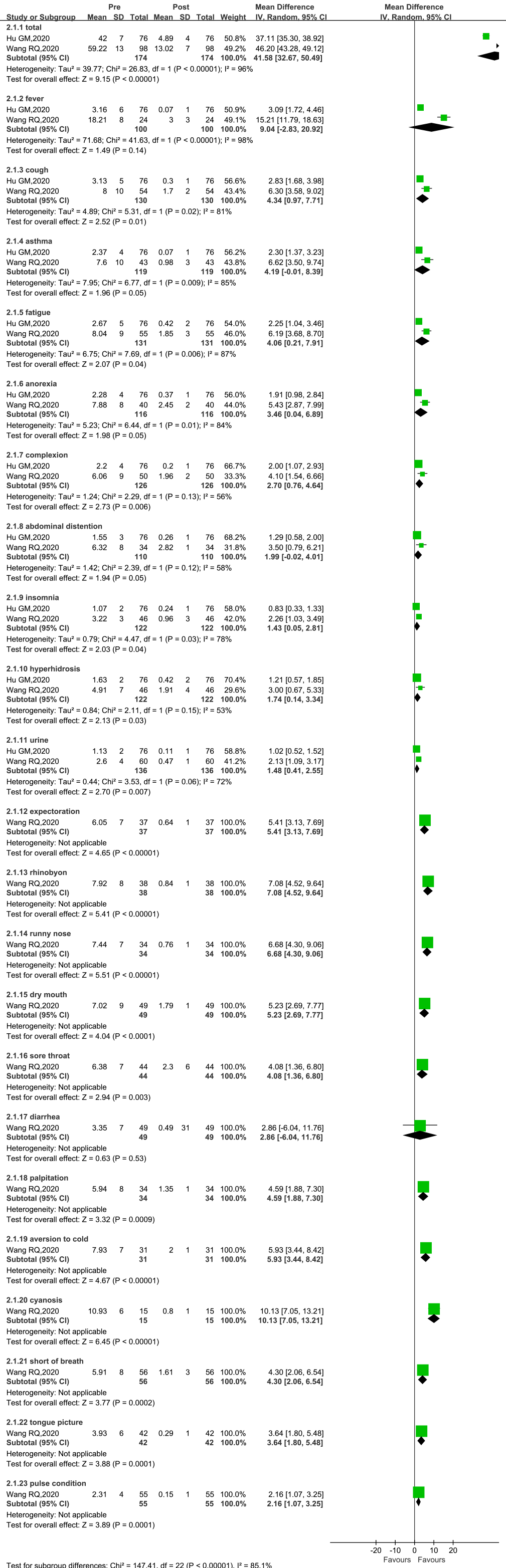

Supplement: Supplementary file 6 [file Image2.TIF]

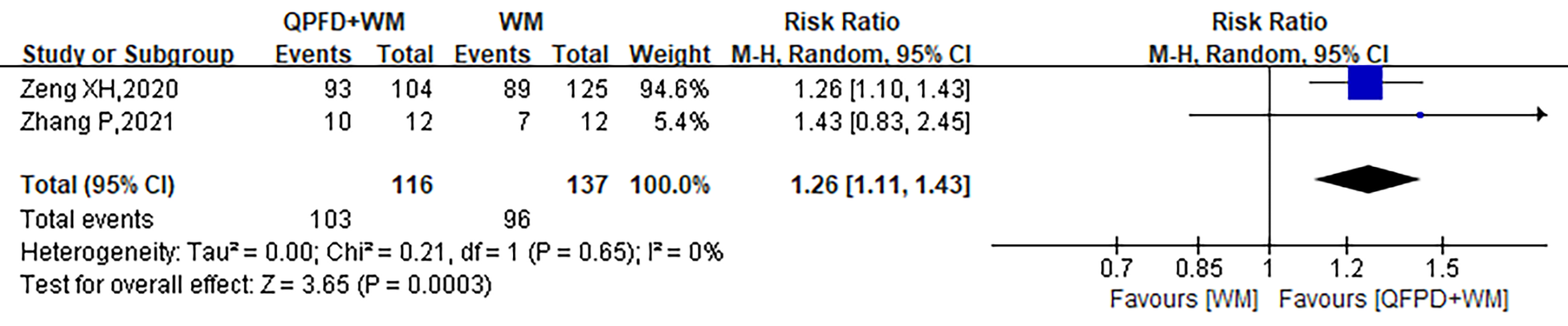

Supplement: Supplementary file 7 [file Image11.TIF]

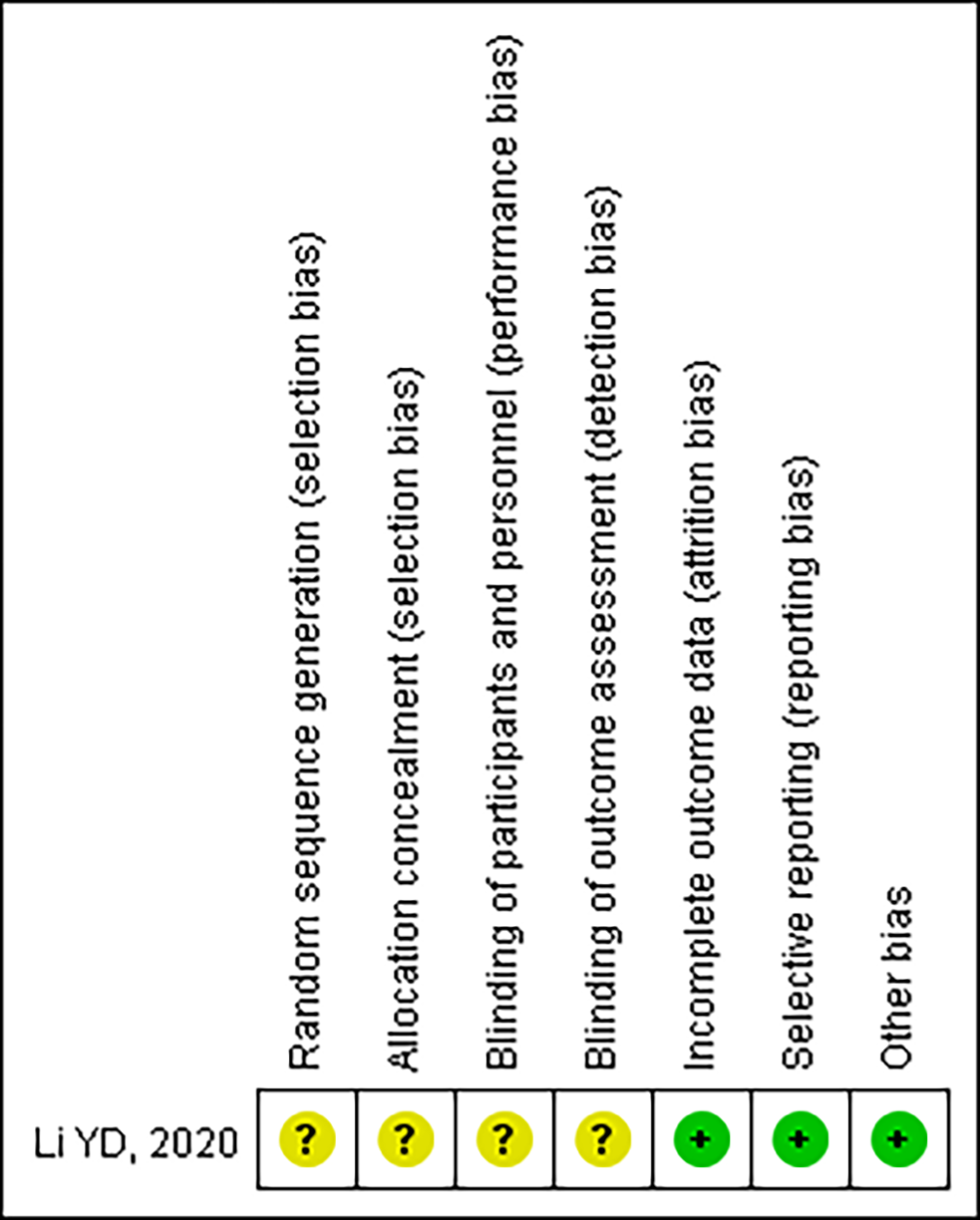

Supplement: Supplementary file 8 [file Image1.TIF]

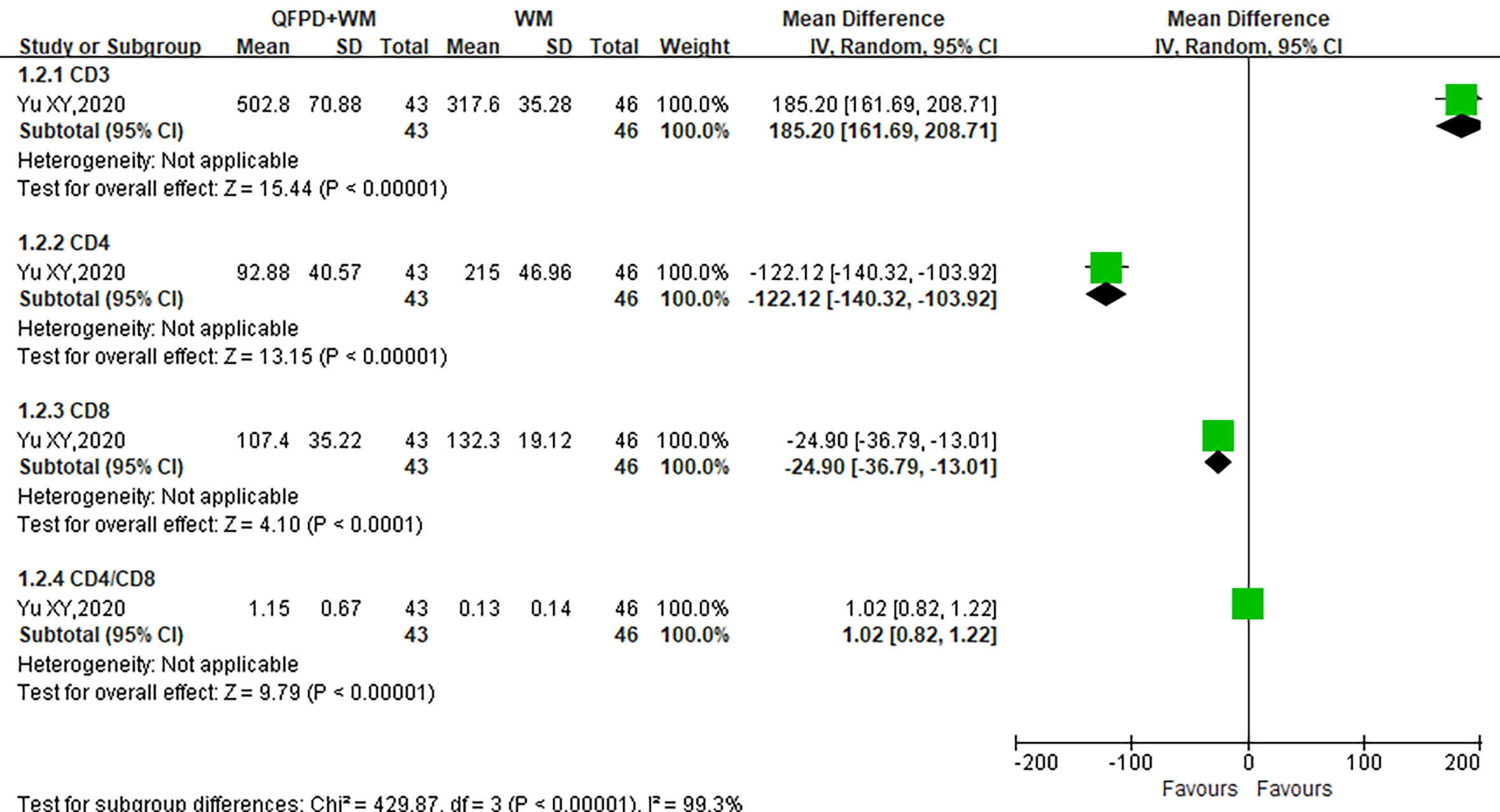

Supplement: Supplementary file 10 [file Image7.TIF]

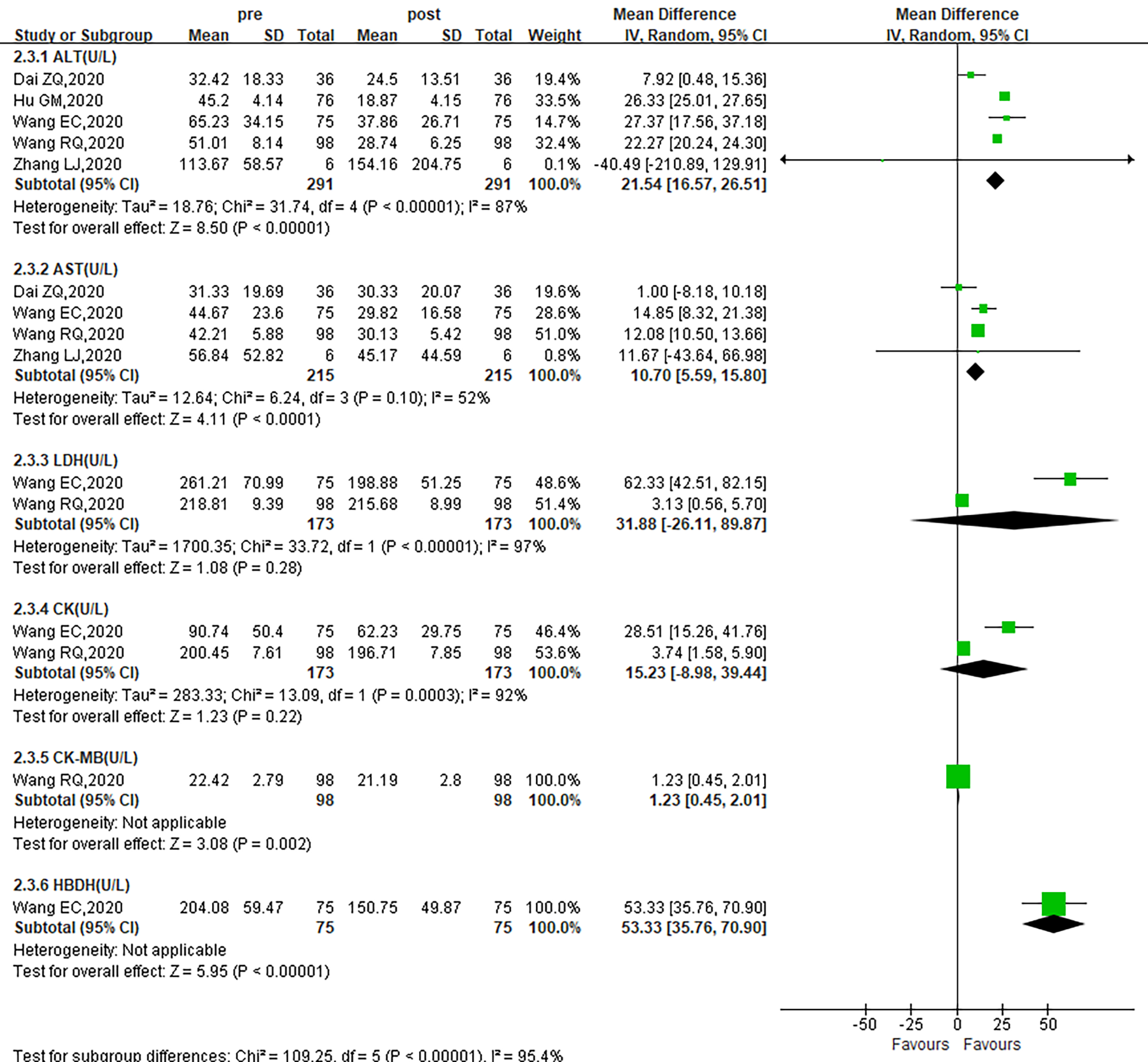

Supplement: Supplementary file 15 [file Image8.TIF]

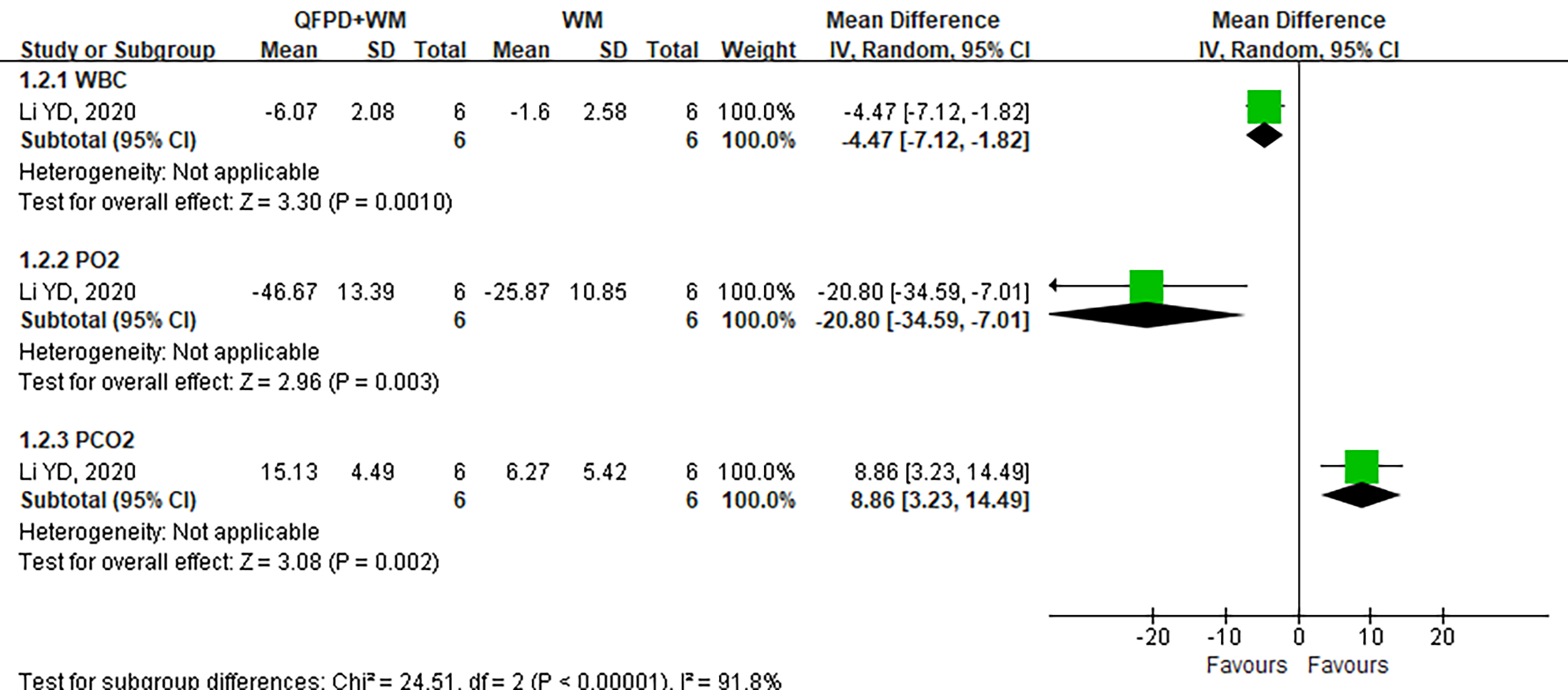

Supplement: Supplementary file 16 [file Image5.TIF]
