# Supplementary material for: Efficacy and Safety of Qingfei Paidu Decoction for Treating COVID-19: A Systematic Review and Meta-Analysis
Source: Front Pharmacol. 2021 Aug 12;12:688857. doi: 10.3389/fphar.2021.688857 (PMC8387832; doi:10.3389/fphar.2021.688857)
Supplement: Supplementary file 2 [file Table1.docx]

**Table S1. Search strategy**

| Database | Searchs | Number |
| --- | --- | --- |
| WHO COVID-19 database* | qingfei paidu “title, abstract,subject” | 49 |
| the Living Overview of the Evidence | qingfei paidu | 14 |
| PubMed | qingfei paidu [Title/Abstract] | 23 |
| China National Knowledge Infrastructure | 主题="清肺排毒汤" | 128 |
| WANFANG | 主题="清肺排毒汤" | 129 |
| Chinese Biomedical Database | "清肺排毒汤"[常用字段:智能] | 138 |
| Chinese Medical Journal Network | "清肺排毒汤" | 2 |

*WHO COVID-19 database included 26 databases published in different languages and grey literature evidence sources around the world: Medline (Ovid and PubMed), PubMed Central, Embase, CAB Abstracts, Global Health, PsycInfo, Cochrane Library, Scopus, Academic Search Complete, Africa Wide Information, CINAHL, ProQuest Central, SciFinder, the Virtual Health Library, LitCovid, WHO covid-19 website, CDC covid-19 website, Eurosurveillance, China CDC Weekly, Homeland Security Digital Library, ClinicalTrials.gov, bioRxiv (preprints), medRxiv (preprints), chemRxiv (preprints), and SSRN (preprints).
