# Supplementary material for: Efficacy and Safety of Qingfei Paidu Decoction for Treating COVID-19: A Systematic Review and Meta-Analysis
Source: Front Pharmacol. 2021 Aug 12;12:688857. doi: 10.3389/fphar.2021.688857 (PMC8387832; doi:10.3389/fphar.2021.688857)
Supplement: Supplementary file 11 [file Table2.docx]

**Table S2. The reasons and lists of studies excluded in full-text screen**

| Study | Study design | Reason for Exclude |
| --- | --- | --- |
| (Li et al., 2020a) | real-world study | multi traditional Chinese medicine |
| (Jang et al., 2021) | observational study | wrong treatment |
| (Yu et al., 2020a) | retrospective study | multi traditional Chinese medicine |
| (Cheng et al., 2020) | case report | wrong treatment |
| (Lai et al., 2020) | retrospective study | wrong treatment |
| (Jiang et al., 2020) | retrospective study | wrong treatment |
| (Liu et al., 2020a) | retrospective study | multi traditional Chinese medicine |
| (Tian et al., 2020) | retrospective study | wrong treatment |
| (Zhong, 2020) | retrospective study | multi traditional Chinese medicine |
| (Wang, 2021) | retrospective study | wrong design |

**Reference**

Cheng, Y.F., Xia, Y.H., Zhou, D.Y., et al. (2020). Syndrome Differentiation-based Treatment of Novel Coronavirus Pneumonia in Bozhou, China: An Analysis of 84 cases. *Journal of Anhui University of Chinese Medicine* 39(2)**,** 9-11.

Lai, H., Yin, W.X., Zhao, F.L., et al. (2020). Effect of self-developed Fuzhengbixie Formula and Qingfeipaidu Mixture on the prevention and treatment of the coronavirus disease 2019. *China Pharmaceuticals* 29(07)**,** 9-11.

Li, H., Lv , W.L., Sun, Y.N., et al. (2020a). Clinical efficacy of traditional Chinese medicine among 749 patients with COVID-19: A real-world study. *China Journal of Traditional Chinese Medicine and Pharmacy* 35(06)**,** 3194-3198.

Liu, J., Yang, D.M., Zhen, L., et al. (2020a). Clinical characteristics of 22 cases of COVID-19 and analysis of the combination of Chinese and Western Medicine. *Journal of Mudanjiang Medical University* 41(04)**,** 42-44+86.

Jang, S., Kim, D., Yi, E., Choi, G., Song, M., and Lee, E.K. (2021). Telemedicine and the Use of Korean Medicine for Patients With COVID-19 in South Korea: Observational Study. *JMIR Public Health Surveill* 7(1)**,** e20236.

Jiang, H., Bai, J., Huang, H., et al. (2020). Clinical investigation and analysis of 53 cases of COVID‐19 patients with traditional Chinese medicine.doi:10.3760/cma.j.cmcr.2020.e00053. *Chinese Medicine Case Repository* 2.

Yu, H.Y., Ren, X.H., Qi, X.X., et al. (2020a). Efficacy Study of Arbidol, Qingfei Paidu Decoction, Lianhua Qingwen Capsule, and Jinye Baidu Granule in the Treatment of Mild/Moderate COVID-19 in a Fangcang Shelter Hospital *Pharmacology and Clinics of Chinese Materia Medica* 36(06)**,** 2-6.

Tian, Z.H., Wu, B., Xiang, J.J., et al. (2020). Clinical Study on the Treatment of Novel Coronavirus Pneumonia by Integrated Traditional Chinese and Western Medicine. *Hebei Journal of Traditional Chinese Medicine* 42(08)**,** 1125-1128.

Zhong, G.F. (2020). Clinical retrospective analysis of integrated traditional Chinese and western medicine in the treatment of new coronavirus pneumonia. *Health Guide* (27)**,** 60.

Wang, Y.P. (2021). Efficacy and safety of Qingfei Paidu Decoction (Granules) in the treatment of COVID-19. *Chinese Clinical Trial Registry.*[*http://www.chictr.org.cn/showprojen.aspx?proj=120429*](http://www.chictr.org.cn/showprojen.aspx?proj=120429)*.*
