# Supplementary material for: Efficacy and Safety of Qingfei Paidu Decoction for Treating COVID-19: A Systematic Review and Meta-Analysis
Source: Front Pharmacol. 2021 Aug 12;12:688857. doi: 10.3389/fphar.2021.688857 (PMC8387832; doi:10.3389/fphar.2021.688857)
Supplement: Supplementary file 12 [file Table5.docx]

**Table S5. Quality assessment of studies according to the ‘Quality assessment for Before-After (Pre-Post) Studies with No Control Group**

| Study | 1.Study  question | 2. Eligibility  criteria | 3.Representative of populations of interest | 4.Participants  enrolled | 5. Sample  size | 6.Intervention | 7. Outcome  measures | 8. Blinding | 9. Follow-up | 10.Statistical  analysis | 11. Multiple outcome measures | 12. Group-level interventions and individual-level outcome  effects | Overall  quality |
| --- | --- | --- | --- | --- | --- | --- | --- | --- | --- | --- | --- | --- | --- |
| (Hu et al., 2020) | Yes | Yes | Yes | Yes | No | Yes | Yes | CD | NA | Yes | No | NA | Fair |
| (Dai et al., 2020) | Yes | Yes | Yes | Yes | No | Yes | Yes | CD | NA | Yes | No | NA | Fair |
| (Meng et al., 2020) | Yes | Yes | Yes | Yes | Yes | No | No | CD | NA | Yes | No | NA | Poor |
| (Liu et al., 2020b) | No | Yes | Yes | Yes | No | Yes | Yes | CD | NA | No | No | NA | Poor |
| (Wang et al., 2020a) | Yes | Yes | Yes | Yes | No | Yes | No | CD | NA | Yes | No | NA | Poor |
| (Wang et al., 2020b) | Yes | Yes | Yes | Yes | No | Yes | Yes | CD | NA | Yes | No | NA | Fair |
| (Zhang et al., 2020) | Yes | Yes | Yes | Yes | Yes | Yes | No | CD | NA | Yes | No | NA | Fair |

*CD: cannot determine; NA: not applicable; NR: not report.

Question 5 was met whenever sample size calculation was explicitly reported, or more than 100 participants were enrolled.

Dai, Z.Q., Jiang, S.H., Liu, T., and Song, G.L. (2020). Novel coronavirus pneumonia treated by Qingfei Paidu decoction: a clinical analysis of 36 cases. *J Guizhou Univ Tradit Chin Med* 42(6)**,** 34-38.

Hu, G.M., He, Z.X., Sun, Q.L., Wan, B.B., Li, Y.B., Gao, J.Y., et al. (2020). Preliminary study on the clinical efficacy of "Qingfei Paidu granules" in treating novel coronavirus pneumonia *Tianjin J Tradit Chin Med* 37(9)**,** 999-1004.

Liu, L.X., Zheng, Y.F., Yang, J., Li, W.W., Lv, J.J., and Fan, C.X. (2020b). Clinical observation on 13 cases of ordinary COVID-19 treated by integrated traditional Chinese and western medicine *Zhejiang J Integr Tradit West Med* 30(5)**,** 349-351.

Meng, J.H., He, Y., Chen, X., Gao, Q., Chen, Y.G., and An, J. (2020). A retrospective study on the treatment of COVID-19 type common/ type severe with Qingfei Paidu decoction. *Chin J Hosp Pharm* 40(20)**,** 2152-2157.

Wang, E.C., Tang, L., Xu, K., and Feng, Q.S. (2020a). Efficacy evaluation of Qingfeipaidu Decoction in the treatment of 75 cases of mild and common type of COVID-19 with enzymatic index. *Pharm and Clin of Chin Materia Medica* 11(1)**,** 3-5.

Wang, R.Q., Yang, S.J., Xie, C.G., Shen, Q.L., Li, M.Q., Lei, X., et al. (2020b). Clinical observation on Qingfei Paidu decoction in treating COVID-19. *Pharm and Clin of Chin Materia Medica* 36(1)**,** 13-18.

Zhang, L.J., Fan, H., Chen, R., Zhu, X.W., Wang, W.Z., Cui, D.D., et al. (2020). Discussion on the rational application of Qingfei Paidu decoction from clinical practice *J Tradi Chin Med* 61(18)**,** 1573-1577.
