# Supplementary material for: Efficacy and Safety of Qingfei Paidu Decoction for Treating COVID-19: A Systematic Review and Meta-Analysis
Source: Front Pharmacol. 2021 Aug 12;12:688857. doi: 10.3389/fphar.2021.688857 (PMC8387832; doi:10.3389/fphar.2021.688857)
Supplement: Supplementary file 13 [file Table3.docx]

**Table S3.** Summary table of Qingfei Paidu Decoction

| Study | Source | Species, concentration | Quality control reported? (Y/N) | Chemical analysis reported? (Y/N) |
| --- | --- | --- | --- | --- |
| (Li and Zhang, 2020) | Not reported | *Herba Ephedrae*, 9g  *Radixet Rhizoma Glycyrrhizae Praeparata cum Melle*, 6g  *Semen Armeniacae Amarum*, 9g  *Gypsum Fibrosum*, 15-30g (decocted first)  *Ramulus Cinnamomi,* 9g  *Rhizoma Alismatis*, 9g  *Polyporus*, 9 g  *Rhizoma Atractylodis Macrocephalae*, 9g  *Poria*, 15g  *Radix Bupleuri*, 16g  *Radix Scutellariae*, 6g  *Rhizoma Pinelliae Praeparatum*, 9g  *Rhizoma Zingiberis Recens*, 9g  *Radix et Rhizoma Asteris*, 9g  *Flos Farfarae*, 9g  *Rhizoma Belamcandae*, 9g  *Radix et Rhizoma Asari*, 6g  *Rhizoma Dioscoreae*, 12g  *Fructus Aurantii Immaturus*, 6g  *Pericarpium Citri Reticulatae*, 6g  *Herba Agastachis*, 9g. | N | N |
| (Zhang et al., 2020a) | 15 Hospitals of Hubei Province in China | Not reported | N | N |
| (Shi et al., 2020) | 54 Hospitals of 9 Provinces in China | Same as *Li and Zhang, 2020* | Y - The quality of the herbs conformed to the criteria set by the 2015 Chinese Pharmacopoeia. Hazardous substances, including pesticide residues, heavy metals, and  microbial contamination, were detected in all herbs to ensure safety, and the results met the criteria in China. QFPD was prepared by a pharmacist according to the standardized procedure in each hospital. | N |
| (Xin et al., 2020) | Xiangyang No. 1 People’s Hospital | *Rhizoma Zingiberis Recens*, 15g  *Gypsum Fibrosum,* 10/30g (30g is for patients with fever)  Other species and concentration of herbs are same as *Li and Zhang, 2020* | Y - All procedures from the purchase of raw materials (batch numbers were reported) for mixture and boiling were performed by the hospital pharmacy. | N |
| (Zeng et al., 2020) | Beijing Tongren Hospital, Capital Medical University | *Radix et Rhizoma Asari*, 3g  *Gypsum Fibrosum*, 30g  Other species and concentration of herbs are same as *Li and Zhang, 2020.* | N | N |
| (Li et al., 2020) | The Third People’s Hospital of Hubei Province | Same as *Li and Zhang, 2020* | N | N |
| (Sun et al., 2020) | 5 Module Hospitals of Hubei Province | Same as *Li and Zhang, 2020* | N | N |
| (Yu et al., 2020) | Renming Hospital of Wuhan University (Hubei General Hospital) | Same as *Li and Zhang, 2020* | N | N |
| (Zhang and Pan, 2021) | Tongji Hospital, Tongji Medical College of Huazhong University of Science & Technology | *Gypsum Fibrosum*, 30g  *Herba Ephedrae* was removed.  Other species and concentration of herbs are same as *Li and Zhang, 2020.* | N | N |
| (Dai et al., 2020) | Ezhou Central Hospital of Hubei Province | Same as *Li and Zhang, 2020* | N | N |
| (Hu et al., 2020) | PuraPharm International Co., Ltd. | Same as *Li and Zhang, 2020* | N | N |
| (Liu et al., 2020) | Zhejiang Benefit Pharmaceutical Co., Ltd. | *Radix Bupleuri*, 9g  *Gypsum Fibrosum*, 30g  Other species and concentration of herbs are same as *Li and Zhang, 2020.* | N | N |
| (Meng et al., 2020) | Wuhan Third Hospital (Tongren Hospital of Wuhan University) | Not reported | N | N |
| (Wang et al., 2020a) | TCM Hospital of Southwest Medical University | Same as *Li and Zhang, 2020* | N | N |
| (Wang et al., 2020b) | 16 Hospitals of Sichuan Province | Same as *Li and Zhang, 2020* | N | N |
| (Zhang et al., 2020b) | West Court, Wuhan Union Hospital | *Radixet Rhizoma Glycyrrhizae Praeparata* cum Melle, 9g  *Gypsum Fibrosum*, 30g  *Herba Agastachis* was removed.  Other species and concentration of herbs are same as *Li and Zhang, 2020.* | N | N |

***Reference***

Dai, Z.Q., Jiang, S.H., Liu, T., and Song, G.L. (2020). Novel coronavirus pneumonia treated by Qingfei Paidu decoction: a clinical analysis of 36 cases. *J Guizhou Univ Tradit Chin Med* 42(6)**,** 34-38.

Hu, G.M., He, Z.X., Sun, Q.L., Wan, B.B., Li, Y.B., Gao, J.Y., et al. (2020). Preliminary study on the clinical efficacy of "Qingfei Paidu granules" in treating novel coronavirus pneumonia *Tianjin J Tradit Chin Med* 37(9)**,** 999-1004.

Li, K.Y., An, W., Xia, F., Chen, M., Yang, P., Liao, Y.L., et al. (2020). Observation on clinical effect of modified Qingfei Paidu Decoction in treatment of COVID-19. *Chin Tradit Herbal Drugs* 51(8)**,** 2046-2049.

Li, Y.D., and Zhang, W.J. (2020). Evaluation on the Clinical Effect of Traditional Chinese Medicine and Western Medicine Regimens on COVID-19. *Guangming J Chin Med* 35(9)**,** 1273-1275.

Liu, L.X., Zheng, Y.F., Yang, J., Li, W.W., Lv, J.J., and Fan, C.X. (2020). Clinical observation on 13 cases of ordinary COVID-19 treated by integrated traditional Chinese and western medicine *Zhejiang J Integr Tradit West Med* 30(5)**,** 349-351.

Meng, J.H., He, Y., Chen, X., Gao, Q., Chen, Y.G., and An, J. (2020). A retrospective study on the treatment of COVID-19 type common/ type severe with Qingfei Paidu decoction. *Chin J Hosp Pharm* 40(20)**,** 2152-2157.

Shi, N.N., Liu, B., Liang, N., Ma, Y., Ge, Y.W., Yi, H.G., et al. (2020). Association between early treatment with Qingfei Paidu decoction and favorable clinical outcomes in patients with COVID-19: a retrospective multicenter cohort study. *Pharmacol Res* 161(105290)**.**

Sun, Y.N., Lv, W.L., Li, H., Xiao, Y., Yang, M., Yang, H.J., et al. (2020). Multi-center clinical research of Qingfei Paidu decoction in 295 cases in the treatment of COVID-19. *J Shandong Univ Health Sci***,** 1-6.

Wang, E.C., Tang, L., Xu, K., and Feng, Q.S. (2020a). Efficacy evaluation of Qingfeipaidu Decoction in the treatment of 75 cases of mild and common type of COVID-19 with enzymatic index. *Pharm and Clin of Chin Materia Medica* 11(1)**,** 3-5.

Wang, R.Q., Yang, S.J., Xie, C.G., Shen, Q.L., Li, M.Q., Lei, X., et al. (2020b). Clinical observation on Qingfei Paidu decoction in treating COVID-19. *Pharm and Clin of Chin Materia Medica* 36(1)**,** 13-18.

Xin, S., Cheng, X., Zhu, B., Liao, X., Yang, F., Song, L., et al. (2020). Clinical retrospective study on the efficacy of Qingfei Paidu decoction combined with Western medicine for COVID-19 treatment. *Biomed Pharmacother* 129**,** 110500.

Yu, X.Y., Zhang, S., Yan, F.F., and Su, D.Z. (2020). Comparison of clinical efficacy of Qingfei Paidu decoction combined with western medicine in 43 cases and single western medicine in 46 cases in the treatment of COVID-19 *J Shandong Univ (Health Sciences)* 58(12)**,** 47-53.

Zeng, X.H., Ma, W.H., and Wang, J. (2020). Effect of Qingfei Paidu decoction on clinical efficacy of COVID-19 pneumonia with phlegm heat blocking lung. *Med J West China* 32(12)**,** 1799-1801+1806.

Zhang, L.H., Zheng, X., Bai, X.K., Wang, Q., Chen, B.W., Wang, H.B., et al. (2020a). Association between Use of Qingfei Paidu Tang and Mortality in Hospitalized Patients with COVID-19: A national retrospective registry study. *medRxiv***.**

Zhang, L.J., Fan, H., Chen, R., Zhu, X.W., Wang, W.Z., Cui, D.D., et al. (2020b). Discussion on the rational application of Qingfei Paidu decoction from clinical practice *J Tradi Chin Med* 61(18)**,** 1573-1577.

Zhang, P., and Pan, G.T. (2021). Clinical study of Qingfei Paidu Decoction on improving inflammatory cytokines in critical patients with COVID-19. *Modern Tradi Chin Medica Materia Medica-World Sci and Techno[J/OL]* 1-5.
