# Supplementary material for: Efficacy and Safety of Qingfei Paidu Decoction for Treating COVID-19: A Systematic Review and Meta-Analysis
Source: Front Pharmacol. 2021 Aug 12;12:688857. doi: 10.3389/fphar.2021.688857 (PMC8387832; doi:10.3389/fphar.2021.688857)
Supplement: Supplementary file 14 [file Table4.docx]

**Table S4.** Risk of bias of included non-randomized control studies

| Study | 1.Representativeness of the Exposed Cohort | 2.Selection of the Non-Exposed Cohort | 3.Ascertainment of Exposure | 4.Demonstration That Outcome of Interest Was Not Present at Start of Study | 5.Comparability of Cohorts on the Basis of the Design or Analysis | 6.Assessment of Outcome | 7. Was Follow-Up Long Enough for Outcomes to Occur | 8.Adequacy of Follow Up of Cohorts | Total |
| --- | --- | --- | --- | --- | --- | --- | --- | --- | --- |
| (Xin et al., 2020) | 1 | 1 | 1 | 0 | 2 | 0 | 0 | 1 | 6 |
| (Zeng et al., 2020) | 1 | 1 | 1 | 0 | 2 | 0 | 0 | 1 | 6 |
| (Li et al., 2020b) | 1 | 1 | 1 | 0 | 2 | 0 | 0 | 1 | 6 |
| (Yu et al., 2020b) | 1 | 1 | 1 | 0 | 2 | 0 | 0 | 1 | 6 |
| (Sun et al., 2020) | 1 | 1 | 1 | 1 | 2 | 1 | 0 | 1 | 8 |
| (Shi et al., 2020) | 1 | 1 | 1 | 1 | 1 | 1 | 1 | 0 | 7 |
| (Zhang et al., 2020) | 1 | 1 | 1 | 1 | 2 | 1 | 0 | 1 | 8 |
| (Zhang and Pan, 2021) | 1 | 1 | 1 | 0 | 2 | 0 | 0 | 1 | 6 |

**Reference**

Li, K.Y., An, W., Xia, F., Chen, M., Yang, P., Liao, Y.L., et al. (2020b). Observation on clinical effect of modified Qingfei Paidu Decoction in treatment of COVID-19. *Chin Tradit Herbal Drugs* 51(8)**,** 2046-2049.

Shi, N.N., Liu, B., Liang, N., Ma, Y., Ge, Y.W., Yi, H.G., et al. (2020). Association between early treatment with Qingfei Paidu decoction and favorable clinical outcomes in patients with COVID-19: a retrospective multicenter cohort study. *Pharmacol Res* 161(105290)**.**

Sun, Y.N., Lv, W.L., Li, H., Xiao, Y., Yang, M., Yang, H.J., et al. (2020). Multi-center clinical research of Qingfei Paidu decoction in 295 cases in the treatment of COVID-19. *J Shandong Univ Health Sci***,** 1-6.

Xin, S., Cheng, X., Zhu, B., Liao, X., Yang, F., Song, L., et al. (2020). Clinical retrospective study on the efficacy of Qingfei Paidu decoction combined with Western medicine for COVID-19 treatment. *Biomed Pharmacother* 129**,** 110500.

Yu, X.Y., Zhang, S., Yan, F.F., and Su, D.Z. (2020b). Comparison of clinical efficacy of Qingfei Paidu decoction combined with western medicine in 43 cases and single western medicine in 46 cases in the treatment of COVID-19 *J Shandong Univ (Health Sciences)* 58(12)**,** 47-53.

Zeng, X.H., Ma, W.H., and Wang, J. (2020). Effect of Qingfei Paidu decoction on clinical efficacy of COVID-19 pneumonia with phlegm heat blocking lung. *Med J West China* 32(12)**,** 1799-1801+1806.

Zhang, L.J., Fan, H., Chen, R., Zhu, X.W., Wang, W.Z., Cui, D.D., et al. (2020). Discussion on the rational application of Qingfei Paidu decoction from clinical practice *J Tradi Chin Med* 61(18)**,** 1573-1577.

Zhang, P., and Pan, G.T. (2021). Clinical study of Qingfei Paidu Decoction on improving inflammatory cytokines in critical patients with COVID-19. *Modern Tradi Chin Medica Materia Medica-World Sci and Techno* 1-5.
